# Metabonomic analysis of follicular fluid in patients with diminished ovarian reserve

**DOI:** 10.3389/fendo.2023.1132621

**Published:** 2023-02-27

**Authors:** Jianan Li, Zhourui Zhang, Yiqiu Wei, Pujia Zhu, Tailang Yin, Qiongqiong Wan

**Affiliations:** ^1^ Reproductive Medicine Center, Renmin Hospital of Wuhan University, Wuhan, Hubei, China; ^2^ The Institute for Advanced Studies, Wuhan University, Wuhan, Hubei, China

**Keywords:** diminished ovarian reserve, follicular fluid, metabonomics, oocytes, embryos, amino acids, steroids

## Abstract

**Background:**

Ovarian reserve is an important factor determining female reproductive potential. The number and quality of oocytes in patients with diminished ovarian reserve (DOR) are reduced, and even if *in vitro* fertilization-embryo transfer (IVF-ET) is used to assist their pregnancy, the clinical pregnancy rate and live birth rate are still low. Infertility caused by reduced ovarian reserve is still one of the most difficult clinical problems in the field of reproduction. Follicular fluid is the microenvironment for oocyte survival, and the metabolic characteristics of follicular fluid can be obtained by metabolomics technology. By analyzing the metabolic status of follicular fluid, we hope to find the metabolic factors that affect the quality of oocytes and find new diagnostic markers to provide clues for early detection and intervention of patients with DOR.

**Methods:**

In this research, 26 infertile women with DOR and 28 volunteers with normal ovarian reserve receiving IVF/ET were recruited, and their follicular fluid samples were collected for a nontargeted metabonomic study. The orthogonal partial least squares discriminant analysis model was used to understand the separation trend of the two groups, KEGG was used to analyze the possible metabolic pathways involved in differential metabolites, and the random forest algorithm was used to establish the diagnostic model.

**Results:**

12 upregulated and 32 downregulated differential metabolites were detected by metabolic analysis, mainly including amino acids, indoles, nucleosides, organic acids, steroids, phospholipids, fatty acyls, and organic oxygen compounds. Through KEGG analysis, these metabolites were mainly involved in aminoacyl-tRNA biosynthesis, tryptophan metabolism, pantothenate and CoA biosynthesis, and purine metabolism. The AUC value of the diagnostic model based on the top 10 metabolites was 0.9936.

**Conclusion:**

The follicular fluid of patients with DOR shows unique metabolic characteristics. These data can provide us with rich biochemical information and a research basis for exploring the pathogenesis of DOR and predicting ovarian reserve function.

## 1 Introduction

The ovarian reserve reflects the sum of follicles at different stages of development in the female ovary and the ability of these follicles to grow, develop and fertilize. With increasing age, follicles decrease gradually in number and function (1). Diminished ovarian reserve (DOR) refers to a decrease in the number and quality of oocytes, DOR is also currently diagnosed mainly by a decrease in basal sinus follicle count and antimilorphan hormone as well as elevated serum basal follicle stimulating hormone levels. DOR that occurs after the age of 40 is usually physiological, while women’s early experience of DOR leads to an early decline in their reproductive function. The most common clinical manifestations of DOR are menstrual disorder, endocrine disorder, poor response to ovarian stimulation and infertility. If not treated in time, it is likely to progress to premature ovarian failure within a few years (2). The etiology of DOR is complex and mainly includes age, genetics, immunity, psychology, environment and other factors. In most patients, the main reasons are still difficult to determine. Recent studies have shown that even if IVF-ET is selected as a treatment means, the pregnancy rate of DOR patients is still reduced due to their low response to ovulation inducing drugs, and their live birth rate is significantly lower than that of women with normal ovarian reserve (3). In addition, patients with DOR have an increased incidence of hypertension during pregnancy (4) and an increased risk of recurrent abortion and aneuploid blastocysts after IVF (5, 6). Due to the unclear etiology and limited therapeutic effect of DOR, it is still one of the great challenges in the clinical treatment of infertility.

Follicular fluid (FF) is formed by the secretion of granulosa cells, membrane cells and oocytes and the diffusion of plasma components from capillaries to the follicular cavity. Its main components include hormones, growth factors, cytokines, proteins, steroids, amino acids and polysaccharides. As the microenvironment for the growth and development of follicles and oocytes, FF is the medium for the exchange of substances and energy between oocytes and the extracellular environment, and alterations in components in FF can reflect the metabolic level and developmental potential of oocytes (7). Studies also show that changes in follicular fluid metabolites, such as progesterone (8), phosphatidylcholine (9), and high-density lipoproteins (10), have an impact on fertilization and early embryonic development. Therefore, studying changes in metabolites in follicular fluid can reveal the factors that affect the development of oocytes and pregnancy outcome in DOR patients.

Metabonomics is a new omic technology developed after genomics and proteomics, and it can simultaneously qualitatively and quantitatively analyze all low molecular weight metabolites in the samples to be detected. Metabonomics reflects events downstream of gene expression and provides valuable information about cell metabolism. Common methods include nuclear magnetic resonance (NMR), gas chromatography−mass spectrometry (GC−MS), liquid chromatography−mass spectrometry (LC−MS), and capillary electrophoresis mass spectrometry (CE−MS) (11). In recent years, the rapid development of metabonomics has also promoted the study of follicular fluid, which is used to assess the metabolic status of oocytes, study the pathogenesis of diseases and identify potential biomarkers (12). The researchers found that the FF showed different metabolic characteristics at different stages of follicular development (13), and the characteristics of FF metabolism in women of different ages were also different (14). In addition, changes in the metabolic map were also found in FF under the conditions of endometriosis (EMS), polycystic ovary syndrome (PCOS) and other diseases (15, 16). Several targeted metabonomic studies have revealed abnormalities in the metabolism of amino acids and oxidized lipids in the FF of DOR patients, and these differential metabolites are significantly related to the number of oocytes retrieved and embryo quality during IVF-ET, which indicates that the decline in oocyte quality in DOR patients may be involved in the impairment of energy utilization and the increase in oxidative stress. Differential metabolites may be used as biomarkers to predict ovarian reserve and embryonic development (17–19). At present, there are few studies on the nontargeted metabonomics of FF from patients with DOR. In this study, the researchers used nontargeted metabonomics technology (high-performance liquid chromatography−mass spectrometry) to expand the detection range of metabolites in the FF of DOR patients and screen potential biomarkers, which supplemented new data for studying the pathogenesis of DOR and the impact of metabolic changes on IVF-ET outcomes of DOR patients.

## 2 Materials and methods

### 2.1 Sample collection and preparation

This study is a prospective clinical experiment that has been approved by the Ethics Committee of Wuhan University People’s Hospital (WDRY2018-K009), and all subjects signed an informed consent form. The subjects were all IVF or intracytoplasmic sperm injection (ICSI)−assisted pregnancy patients at Wuhan University People’s Hospital from December 2020 to January 2022, and they were divided into an experimental group (DOR group, n=26) and a control group (CON, n=28) according to whether the ovarian reserve was normal.

The inclusion criteria of the DOR group were as follows: 1: 20-40 years old; 2. body mass index (BMI) ≤ 24 kg/m2; and 3. patients meeting the clinical diagnostic criteria of DOR (20): 1) AMH ≤ 1. 1 ng/mL; 2) basic follicle stimulating hormone (bFSH) ≥ 10 IU/L, with or without FSH/luteinizing hormone (LH) ≥ 3.2; and 3) number of follicles in the unilateral basal sinus (bAFC) ≤ 7. Any two of the above three items can be diagnosed as DOR. In the control group, patients with normal ovarian reserve were treated with IVF/ICSI only because of male infertility or oviduct factors (oviduct adhesion, excision, ligation, etc.). The inclusion criteria of the control group were as follows: 1, 20-35 years old; 2. BMI ≤ 24kg/m2; 3, AMH ≥ 2 ng/mL, with or without bAFC ≥ 7; 4. bFSH < 10 IU/L, and estradiol (E2) < 80 pg/mL.

All subjects in this experiment should meet the following exclusion criteria: 1. History of ovarian surgery: cyst stripping, oophorectomy, etc.; 2. EMS, PCOS, ovarian cysts and other diseases that may affect the ovarian reserve function; 3. Received hormone therapy three months before the visit; 4. Any contraindication for ovulation induction therapy; 5. Other systemic abnormalities included hereditary diseases such as chromosome abnormalities, endocrine diseases such as hyperthyroidism and diabetes, infectious diseases such as hepatitis B, HIV and syphilis, and autoimmune diseases such as systemic lupus erythematosus.

### 2.2 Oocyte acquisition and follicular fluid collection

Subjects received an individualized ovarian stimulation protocol according to their ovarian reserve, age, weight and previous ovulation induction. In our reproductive center, the gonadotropin-releasing hormone antagonist protocol (GnRH-ant protocol) and ovarian stimulation protocol under progesterone (PPOS protocol) are used to help patients with DOR. The GnRH-ant protocol: patients begin daily gonadotropin (Gn) on days 2-3 of the menstrual cycle, and follicular development and serum hormone levels are monitored at the same time. When one follicle diameter reaches 12 mm or serum E2 > 300 pg/ml, patients should take GnRH ant 0.25/d; until three follicles have a diameter greater than 18 mm, and 60% of the follicles have a diameter of 16 mm or E2 has no significant increase or decline, they should be injected with HCG6000-10000 IU that night, and after 34-38 hours, they are punctured for oocytes under ultrasonic monitoring. The PPOS protocol is as follows: patients begin daily progesterone (8-10 mg) on days 2-5 of the menstrual cycle, and at the same time, they are injected with FSH or human menopausal gonadotropin (HMG) 150-300 IU/day. The amount of Gn was adjusted according to follicular growth. When one follicle diameter is greater than 17 mm, patients are injected with HCG 6000-10000 IU or GnRH-a 0.2 mg combined with HCG 2000 IU to induce ovulation. Oocytes were retrieved 34-38 h after ovulation induction. Patients who still have more than 2 small follicles (diameter ≤ 8 mm) after oocyte retrieval in the follicular phase, they should continue to take oral Gn, FSH or HMG (150-300 IU) in the luteal phase and adjust the Gn dose 2-3 days later according to follicular development. When at least 2-3 follicles had a diameter greater than 18 mm, and the serum E2 level reached an average of approximately 200 pg/ml for each dominant follicle, HCG (10000 IU) was injected. Oocytes were retrieved 34-38 h after injection.

Only follicular fluid from follicles larger than 18 mm in diameter was collected. The patient’s oocytes were retrieved and placed in the incubator for 4-6 hours, and the follicular fluid was collected in Eppendorf tubes after merging. The collected follicular fluid was centrifuged at 4°C and 15000 rpm for 10 minutes, and the supernatant was collected and frozen at -80°C for the next step of detection.

### 2.3 Embryo quality assessment

The embryo quality was recorded on the third day after fertilization. The evaluation criteria for embryos are as follows: Grade I: the shape of blastomere is regular, the size is uniform, and there is no obvious DNA fragment; Grade II: the size of blastomere is slightly uneven, the shape is slightly irregular, and the fragments are not more than 20%; Grade III: blastomeres vary in size, fragments between 20% to 50%; Grade IV: blastomeres of different sizes, fragments ≥ 50%. High-quality embryos at cleavage stage are defined asfollows: Day3 embryos are Class I~II, and the number of blastomeres is 6~9.

### 2.4 Mass spectrometry detection: sample preparation and spectrum collection

In this study, we used a timsTOF Pro mass spectrometer (Bruker Daltronics, Germany) in combination with an Ultimate 3000 liquid system (Thermo Scientific) for LC−MS analysis. MS data were obtained using a timsTOF Pro mass spectrometer with a TOP 3 data-dependent acquisition (DDA) method. The scanning range of the timsTOF Pro mass spectrometer was 20-1300 Da, and the collision energy of MS/MS was 20 eV. The ESI source conditions were set as follows: capillary voltage=3.6 kV; dry gas flow=10.0 L/min; Spray gas = 2.2 bar; drying temperature=220 °C. Before injecting each mode, we performed external calibration with sodium formate and injected 15 QC samples before FF samples to balance the instrument. During data collection, one QC sample was inserted for every 10 FF samples to monitor the consistency and stability of the whole operation process. The Ultimate 3000 liquid system was mainly used for chromatographic separation. To improve the quality and quantity of identification of hydrophilic and hydrophobic metabolites, two chromatographic separation methods, HILIC and RPLC, were both used for nontargeted metabonomic analysis. In HILIC mode, we used a Waters ACQUITY UPLC BEH amide column (2.1 mm * 100 mm, 1.7 μm), the column temperature was 40 °C, and the injection volume was 5 μL. The mobile phase consisted of A (H2O containing 10 mM ammonium acetate and 0.1% acetic acid) and B (containing 95% ACN and 10 mM ammonium acetate and 0.1% acetic acid), with a constant flow rate of 0.5 mL/min. The chromatographic gradient program was as follows: 0 ~ 1 min, 100% B; 1 ~ 11 min, 60% B; 11~11.5 min, 40% B; 11.5 ~ 12 min, 40% B; 12 ~ 12.1 min, 100% B; 12.1 ~ 18 min, 100% B. In RPLC mode, we used Waters ACQUITY UPLC BEH C18 column (2.1mm * 100mm, 1.7 μm), the column temperature was maintained at 50°C, and the injection volume was 5μ. The mobile phase consisted of A (H2O containing 0.1% formic acid) and B (containing 0.1% formic acid and ACN), with a constant flow rate of 0.4 mL/min. The chromatographic gradient program was as follows: 0 ~ 2 min, 2% B; 2 ~ 17 min, 100% B; 17 ~ 20 min, 100% B; 20 ~ 20.1 min, 2% B; 20.1 ~ 23 min, 2%.

### 2.5 Data processing and statistical analysis

MetaboScape4.0 software was used to process the original data with peak filtering (greater than 5000), calibration, feature matching and standardization to conduct comprehensive molecular feature extraction. After determining the mass to charge ratio and peak intensity of metabolites, we searched the Human metabolome Database (HMDB) and the database established by local standards to retrieve and identify the detected metabolites. Subsequently, the data of all metabolites were normalized on Metaboanalyst 5.0 (www.metaboanalyst.ca). We excluded features with CV values greater than 0.25, filled in the missing value (5.5%) with 1/5 of the positive minimum value, and normalized the data according to the sum. Benjamin Hochberg correction was applied to keep the risk of type I errors below 5%. Using a nonparametric test, we set the fold change (FC)>1.2 and false discovery rate (FDR)<0.05 to screen potential differential metabolites related to DOR. Unsupervised pattern recognition principal component analysis (PCA) and orthogonal partial least squares discriminant analysis (OPLS-DA) were also analyzed on SIMCA software after autoscaling. By reducing the dimensions of the obtained multidimensional data to form a matrix, OPLS-DA could show the separation trend between the two groups more clearly. In addition, the permutation test was used to verify the imitative effect and prediction ability of the OPLS-DA model. Metabolites with first principal component projection (VIP) > 1.0 and P value < 0.05 were considered to be significantly different between the two groups. Kyoto Encyclopedia of Genes and Genomes (KEGG) analysis was conducted to identify pathways that might be involved. Finally, a random forest algorithm was used to establish the diagnostic model.

The baseline characteristics of the subjects were analyzed, and the data are expressed as the mean ± standard deviation (SD). SPSS 25.0 software was used to analyze the normality of the data. Variables with normal or near normal distributions were analyzed by Student’s t test or the chi-square test, and data with nonnormal distribution were analyzed by the Kruskal−Wallis test. The Spearman correlation coefficient between differential metabolites and the ovarian function index was calculated using the psych package in R. Statistical significance was defined as P < 0.05.

## 3 Results

### 3.1 Clinical characteristics of patients with DOR

The patients’ clinical characteristics are showed in **Table 1**. The average age, BMI, years of infertility, fasting blood glucose (FBG) level, serum hormone level and IVF-ET outcome were compared between the DOR and CON groups. Age, bAFC, AMH, and bFSH were significantly different between the two groups (P < 0.001). BMI (P=0.016, P<0.05) and years of infertility (P=0.021, P<0.05) had a slight increase in the DOR group, while bLH, bE2, P and FBG had no differences (P>0.05). Analysis of the results of oocytes obtained and fertilization revealed that the number of oocytes retrieved, MII oocytes, 2PN fertilization and high-quality embryos on the third day in DOR patients were significantly lower than those in CON patients (P<0.001). This shows that the ovarian reserve of patients in DOR group is indeed reduced and our inclusion criteria are reliable. DOR is an age-related infertility, ovarian reserve and oocyte quality decline with advancing age, the difference in age between the DOR group and the control group in the results may be explained by the fact that age is to some extent an etiological or synergistic factor in DOR. To make the study more rigorous, future studies should expand the sample size and distinguish between different age groups before comparing metabolic levels

**Table 1:** The demographic and clinical characteristics of patients with DOR and CON.

Item	DOR group (n=26)	CON group (n=28)	P value
Age (year)	34.04 ± 3.8	30.29 ± 2.62	< 0.001 ***
BMI (kg/m^2^)	21.57 ± 1.43	20.48 ± 1.74	0.016 *
bFSH (mIU/ml)	11.11 ± 4.71	6.60 ± 1.75	< 0.001 ***
bLH (mIU/ml)	4.22 ± 4.36	3.65 ± 1.71	0.52
bE2 (pg/ml)	47.55 ± 30.86	36.02 ± 17.33	0.094
P (ng/ml)	1.49 ± 2.77	0.78 ± 1.03	0.21
bAFC (n)	3.5 ± 1.61	10.18 ± 3.35	< 0.001 ***
AMH (ng/mL)	0.72 ± 0.36	4.70 ± 2.49	< 0.001 ***
FBG (mmol/L)	4.78 ± 0.45	5.02 ± 0.31	0.474
Duration of infertility (year)	3.28 ± 2.77	2.78 ± 2.31	0.021 *
Oocytes retrieved (n)	4.31 ± 2.49	15.07 ± 6.43	< 0.001 ***
MII oocytes (n)	3.12 ± 1.77	10.21 ± 4.83	< 0.001 ***
2PN Fertilizations (n)	2.38 ± 1.68	7.14 ± 4.09	< 0.001 ***
Day3 High-quality embryos (n)	1.04 ± 1.08	4.43 ± 3.56	< 0.001 ***

DOR, Diminished ovarian reserve; NOR, Normal ovarian reserve; BMI, Body mass index; bFSH, basic follicle-stimulating hormone; bLH, basic luteinizing hormone; bE2, basic estrogen; P, progesterone; bAFC, basic antral follicle count; AMH, anti-Mullerian hormone; FBG, fasting blood glucose *P ≤ 0.05 **P ≤ 0.005 ***P ≤ 0.001.

### 3.2 Multivariate analysis of metabolites

In this experiment, we detected metabolites in follicular fluid under HILIC positive and negative ion modes and RPLC positive and negative ion modes. A total of 2897 variable features were detected under HILIC mode, and 3419 features were detected under RPLC mode; a total of 994 metabolites were identified. The Wayne diagram in **Supplementary Figure 1** (**Figure S1**) shows that the metabolites detected in the four modes are very different, which proves that detection in different modes can obtain more information about metabolites.

**Figure 1:**
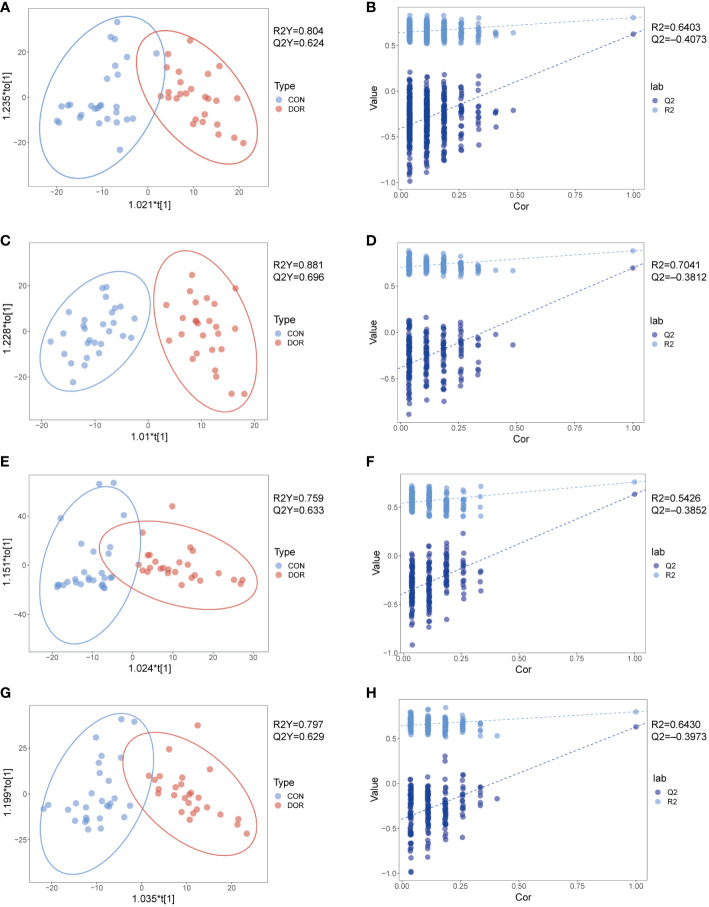
Metabolomic analysis of follicular fluid from patients with diminished ovarian reserved (n = 26, green dots) and healthy controls (n = 28, red dots) under HILIC and RPLC mode. OPLS-DA score plots for positive ionization mode **(A, E)** and negative ionization mode **(C, G)**, the OPLS-DA model’s permutation test for positive ionization mode **(B, F)** and negative ionization mode **(D, H)**. **(A-D)**: HILIC mode; e-h: RPLC mode.

To compare the overall difference in the metabolic spectrum between DOR patients and the control group, PCA and OPLS-DA discriminant models were established based on the metabolite data obtained under four modes. The OPLS-DA discriminant model reveals that the DOR group and control group can be well separated, as shown in **Figures 1A, C, E, G**, the DOR group is all clustered on the right side, the control group is all clustered on the left side, and the values of R2Y and Q2Y are close to 1, which indicates that the difference between the two groups is significant and the prediction ability of the OPLS-DA model is good. As shown in **Figures 1B, D, F, H**, the permutation test shows the value of Q2 and R2 under the four modes, further verifying that the OPLS-DA model has a good imitative effect and prediction ability. In addition, the PCA models also show that the DOR group and control group are distributed in different regions (**Figure S2**), but the two groups are not completely separated in the PCA model, which indicates that the samples have individual differences. In **Figure S2**, the quality control samples (QC) are gathered together, which shows that the stability and repeatability of the detection system is satisfactory, and the OPLS-DA is reliable.

### 3.3 Identification of differential metabolites and analysis of related pathways

**Figure 2:**
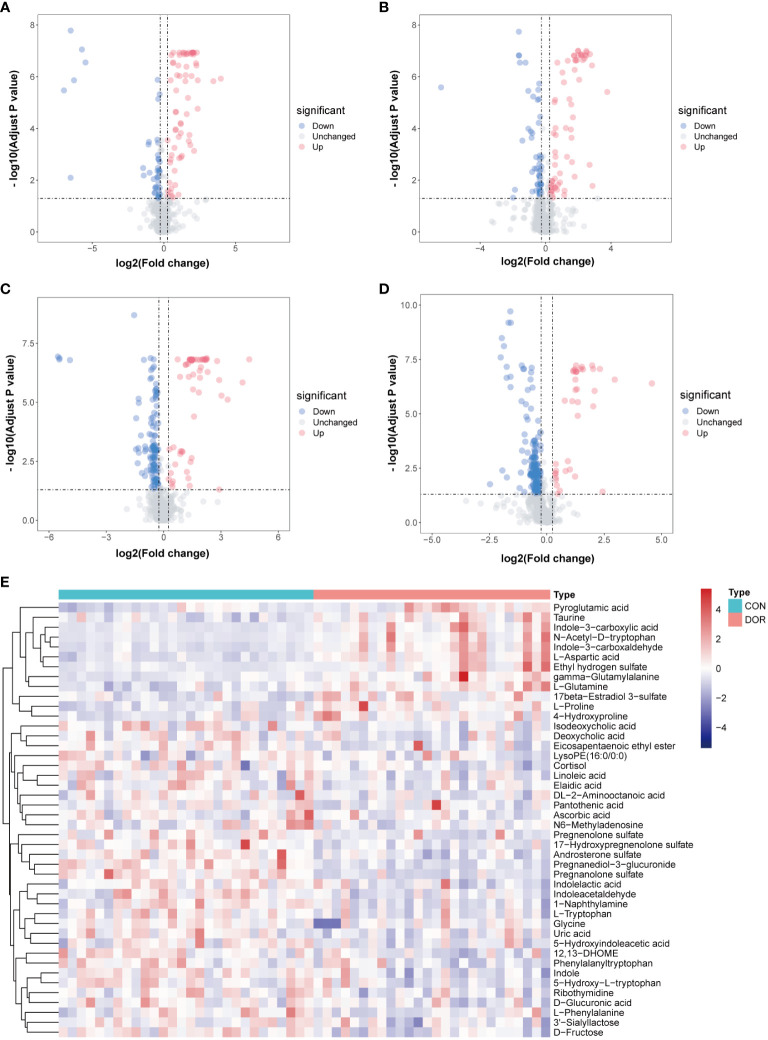
Volcanic maps under different detection modes show the distribution of different features. The blue dot represents the up regulated features while the red dot represents the down regulated. **(A)**: positive ionization mode in HILIC; **(B)**: negative ionization mode in HILIC; **(C)**: positive ionization mode in RPLC; **(D)**: negative ionization mode in RPLC; **(E)**: Heatmap based on 44 metabolites, unsupervised cluster analysis showed that differential metabolites could distinguish DOR patients from healthy subjects.

Univariate analysis was carried out on all features detected in follicular fluid, and a volcano plot is shown in **Figures 2A–D**, downregulated features in DOR samples were clustered on the left, and upregulated features were clustered on the right. A total of 12 upregulated and 32 downregulated metabolites were identified from all differential features. Differential metabolites mainly include amino acids, indoles, nucleosides, organic acids, steroids, phospholipids, fatty acyls and organic oxygen compounds. The VIP value of these differential metabolites in OPLS-DA was greater than 1, and P < 0.05 in univariate analysis (**Supplementary Tables 1**, **2**), which also indicated that they were related to ovarian reserve. In addition, the heatmap also intuitively shows the change in metabolites in DOR patients (**Figure 2E**).

**Figure 3:**
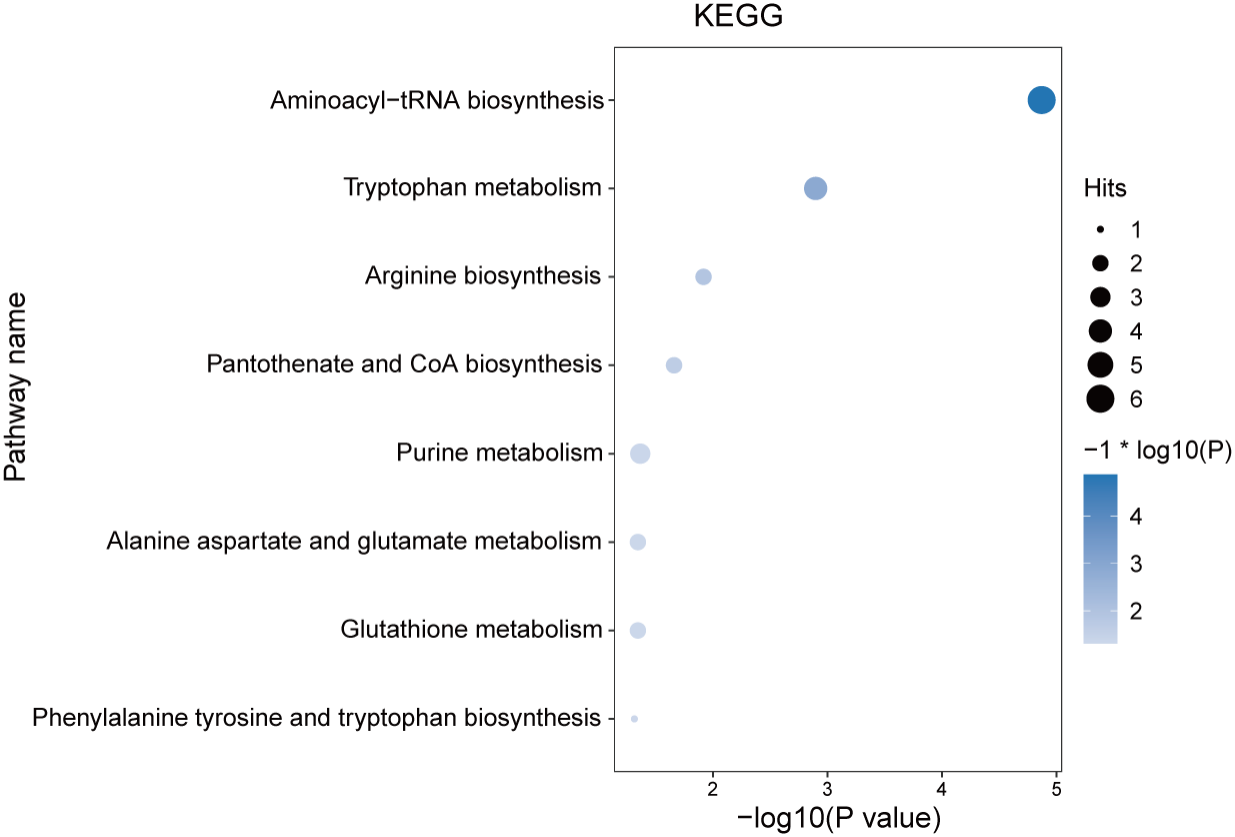
KEGG’s metabolic pathway for differential metabolites. As the bubble color deepens, the P value decreases, and the larger the bubble is, the greater the impact of this pathway on DOR.

According to KEGG analysis, the differential metabolites are mainly involved in aminoacyl-tRNA biosynthesis, tryptophan metabolism, pantothenate and CoA biosynthesis, and purine metabolism (marked in **Figure 3**). These metabolic pathways play an important role in maintaining the normal function of cells and tissues, and the results add new data for exploring the pathogenesis and clinical treatment of DOR.

### 3.4 Correlation analysis between differential metabolites and clinical indicators

**Figure 4:**
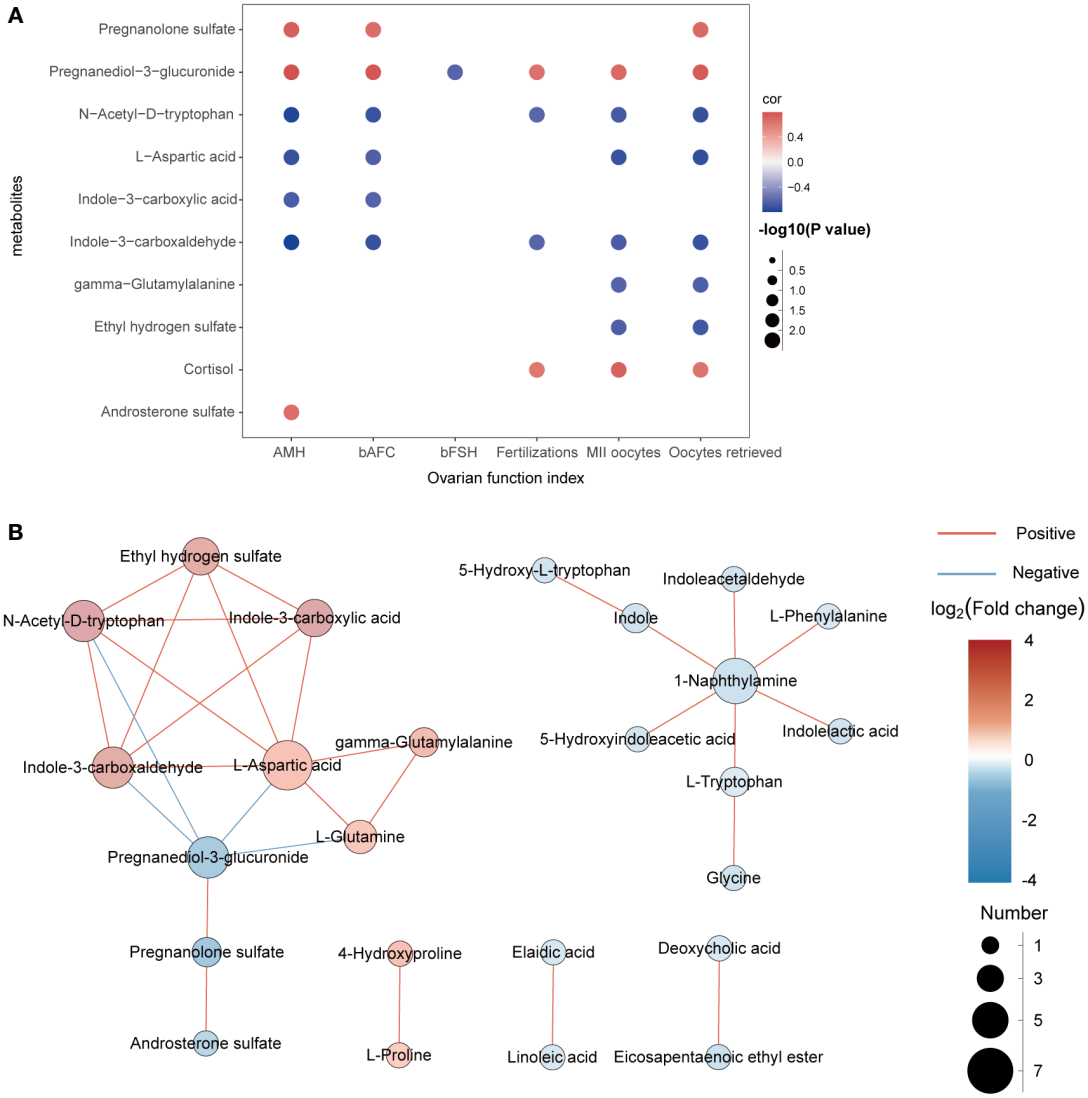
**(A)**: Correlation analysis between differential metabolites and clinical indicators. Red represents positive correlation, while blue represents negative correlation. The darker the color is, the stronger the correlation is. **(B)**: Network diagram of the interactions between metabolites.

In this experiment, Spearman correlation analysis was used to study the relationship between differential metabolites and clinical detection indicators in FF. Ten metabolites with correlation coefficients of R>0.6 and P<0.05 are shown in **Figure 4A**. We found that pregnanediol-3-glucuronide, N-acetyl-D-tryptophan, L-aspartic acid and indole-3-carboxaldehydewere significantly positively correlated with AMH, bAFC, number of oocytes retrieved, MII oocytes, and 2PN fertilizations, while almost all metabolites did not correlate with BMI, bLH, bP, bE2, and duration of infertility (R < 0.3, not shown in the figure). In addition, for further study on how the metabolic profile changed in the FF of DOR patients, the correlation between metabolites is shown in **Figure 4B**.

### 3.5 Establishment of a diagnostic model based on 10 metabolites

**Figure 5:**
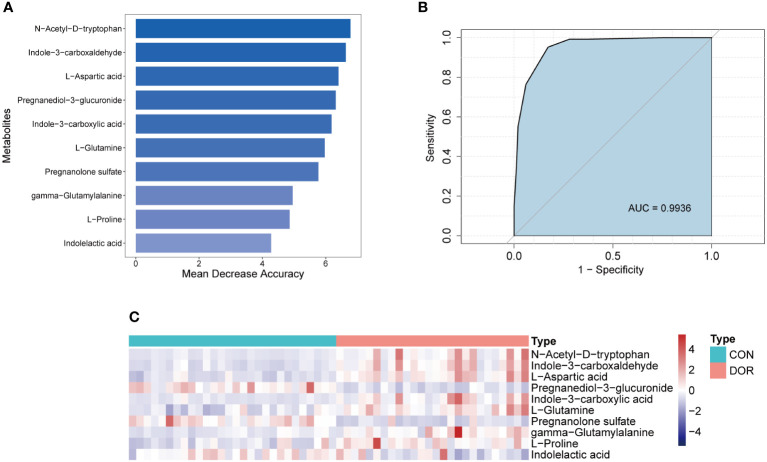
Establishment and test of diagnostic model based on 10 metabolites. **(A)**. Top 10 metabolites selected according to random forest algorithm; **(B)**. ROC curve and AUC value of the combined diagnostic model of the top 10 metabolites; **(C)**. Unsupervised cluster analysis of the top 10 metabolites.

To intervene earlier and more individually in the development of DOR, we used a random forest algorithm to distinguish the DOR groups and the CON groups and finally selected the top 10 metabolites (**Figure 5A**) to be included in the diagnostic model. Using a receiver operating characteristic (ROC) curve to evaluate the diagnostic performance of this model, as shown in **Figure 5B**, the AUC reached a very high value of 0.9936. When all the metabolites were chosen for diagnostic models, the AUC value (AUC=0.9936) was similar to that of the top 10 metabolites. Although the top 20 metabolites had the highest AUC values (AUC=0.9952) when included in the diagnostic model, it was not much higher than 0.9936, so we finally determined the top 10 metabolites for the sake of efficient clinical diagnosis. In addition, unsupervised cluster analysis showed that the top 10 metabolites could distinguish all DOR patients from normal subjects (**Figure 5C**). In summary, these 10 metabolites can be used as promising tools to predict ovarian function.

## 4 Discussion

In this study, we used nontargeted metabonomics to detect the follicular fluid of 54 subjects, and found that there were 12 upregulated and 32 downregulated metabolites in the FF of DOR patients compared with normal women. These metabolites were mainly involved in aminoacyl-tRNA biosynthesis, tryptophan metabolism, pantothenate and CoA biosynthesis, purine metabolism, which are closely related to the number of oocytes and embryo quality. In the following, we discuss in detail the possible mechanisms of differential metabolites.

As an essential amino acid, tryptophan has three metabolic pathways *in vivo*: 1) tryptophan is hydroxylated and decarboxylated to form serotonin and melatonin; 2) tryptophan generates nicotinic acid, nicotinamide adenine dinucleotide (NAD) and nicotinamide adenine dinucleotide phosphate (NADP) through karinurine metabolism; and 3) tryptophan can generate indole, indole pyruvic acid, indole lactic acid, etc., through deamination and decarboxylation. Tryptophan and its metabolites participate in many physiological processes, such as maintaining cell growth and regulating immune function. In recent years, research on tryptophan metabolism in the reproductive field has made some progress. A large number of studies have shown that serotonin, as one of the main metabolites of tryptophan, plays an important role in regulating placental function and fetal development (21). Serotonin can affect the development of oocytes by regulating progesterone secretion by granulosa cells. Animal experiments have also shown that a decrease in serotonin in mouse blood leads to damage to early embryo development (22). Serotonin is the main source of melatonin. The latest evidence showed that human ovarian granulosa cells cultured *in vitro* can express melatonin, and melatonin reduces oxidative stress by improving the mitochondrial function of oocytes (23). In addition, adding an appropriate amount of melatonin during embryo culture improves the rate of high-quality embryos in patients with repeated low-quality embryos on the third day (24), and the quality of embryos and clinical results of patients with repeated cycles after the failure of IVF/ICSI can also be improved (25). Only a small amount of tryptophan is metabolized through the indoleacetic acid pathway, and the role of metabolites generated from this pathway in oocyte and embryo development is still unclear. In this experiment, we detected that tryptophan and its decomposition products 5-hydroxy-L-tryptophan, 5-hydroxyindoleacetic acid (the metabolic end product of serotonin), indole, indoleacetaldehyde, and indoleacetic acid in the FF of DOR patients decreased, and were positively correlated with the number of oocytes, fertilized embryos, and high-quality embryos. The results of this study indicate that the decrease in tryptophan and its decomposition products, especially in the ovary, will affect the quantity and quality of oocytes, and may be detrimental to the early development of embryos. In addition, karinurine metabolism plays a dominant role in tryptophan catabolism, and changes in the concentration of karinurine in the placenta are closely related to the occurrence of several pregnancy complications (26). Recent studies have also shown that the tryptophan-karinurine pathway is abnormally activated in PCOS patients (27). However, in the FF of patients with DOR, we did not detect differential metabolites related to this pathway. Does this indicate that the effect of tryptophan on the development of oocytes and early embryos in DOR patients is through the serotonin and indoleacetic acid metabolic pathways, but not through the karinurine pathway? This need to be further explored.

We also found that other amino acids in FF changed: glycine, phenylalanine and DL-2-aminooctanoic acid were downregulated, and aspartic acid, proline, L-glutamine and pyroglutamate were upregulated. Glycine is a component of the endogenous antioxidant glutathione (GSH), and the reduction may mean that the oocytes are suffering from enormous oxidative stress; therefore, glycine is consumed to synthesize glutathione and resist oxidative stress. Studies have shown that glycine treatment of pig oocytes cultured *in vitro* promoted oocyte maturation by reducing the level of intracellular reactive oxygen species (ROS) and increasing mitochondrial function (28). In addition, glycine also has an effect on mammalian oocyte maturation and early embryo implantation through a volume regulation mechanism (29). L-proline is a key regulator of embryogenesis, placental development and fetal growth. It increased the viability of porcine trophoblastic ectodermal cells by activating the TORC1 signaling pathway (30). Maternal L-proline supplementation could also improve mouse placental development and fetal survival (31). L-glutamine is another important energy substrate that plays a role in the development of preimplantation embryos (32, 33). However, how these two amino acids affect oocyte development is still unclear. We detected changes in amino acids and their derivatives in the FF of DOR patients, and it has been shown that amino acids promote oocyte maturation and early embryonic development by reducing oxidative stress and enhancing mitochondrial function. However, whether amino acids participate in glucose metabolism to regulate cell energy utilization and what kind of synergistic or antagonistic effects they have in DOR patients need further study.

Steroids are another group of metabolites that are significantly altered in the FF of DOR patients. We found that androsterone sulfate, pregnenolone sulfate, pregnanolone sulfate, and 17-hydroxypregnenolone sulfate were decreased; taurine, glucuronic acid and pregnenol-3-glucuronic acid which are closely related to the excretion of gonadal hormones were also downregulated; while 17beta-Estradiol 3-sulfate (E2-3S) was upregulated. Pregnenolone sulfate, 17-hydroxypregnenolone sulfate and androsterone sulfate are the starting points for the synthesis of progesterone, estrogen and cortisol (34). Progesterone is mainly converted into pregnenol-3-glucuronic acid, and estrogen is mainly excreted in the form of sulfate and glucuronic acid. Our results seemed to indicate that compared with normal women, estrogen and progesterone in the FF of DOR patients are decreased. Yang et al. found that genes regulating cholesterol synthesis and transport, such as SCAP, FDFT1, CYP51A1, SRB1 and STARD1 were significantly downregulated in granulosa cells of DOR patients, and serum estradiol and progesterone were significantly lower in DOR groups (35), which complemented our findings. Dehydroepiandrosterone (DHEA) is mainly derived from 17-hydroxypregnenolone sulfate, and study have proved that DHEA treatment improved the clinical outcome of DOR patients receiving IVF-ET (36). DHEA improves the quality of oocytes by increasing the oxidative phosphorylation of mitochondria and reducing the apoptosis of cumulus cells (37, 38). During oocyte activation, DHEA at the normal threshold could significantly improve the fertilization rate and live birth rate (39). Our study found that the downregulation of 17-hydroxypregnenolone sulfate was related to the number of oocytes retrieved, MII oocytes and high-quality embryos, which also implied that 17-hydroxypregnenolone sulfate and DHEA played an important role in the growth and development of oocytes and embryos. At present, the research on metabolites in the steroid group is not perfect, and the metabolic map obtained may not be complete, so more experiments are needed to further understand how the steroid group affects oocyte and embryo development.

The energy required for oocyte growth and meiosis mainly depends on the oxidative phosphorylation of mitochondria. However, the ability of oocytes to ingest and consume glucose is limited, the pyruvate needed for oxidative phosphorylation is mostly supplied by the glycolysis pathway of cumulus cells and granulosa cells (40), and increasing glycolysis in granulosa cells also promotes the activation of primordial follicles (41). In our study, D-fructose, pantothenic acid and 3-sialyllactose were downregulated, which seemed to point to the decreased energy metabolism of cumulus cells and oocytes in DOR patients. Pantothenic acid is necessary for coenzyme A (CoA), which is a cofactor of many enzymes. CoA participates not only in the metabolism of sugar, fat and protein but also in the antioxidation of the body. This study also detected that ascorbic acid was reduced in FF, and the bioactive form of ascorbic acid is vitamin C, which is considered an antioxidant. The results indicated that the follicular microenvironment in patients with DOR seemed to be damaged by oxidative stress, thus adversely affecting the growth and maturation of oocytes.

Some fatty acids, such as linoleic acid, eicosapentaenoic ethyl ester, elaidic acid and 12,13-DHOME, were also downregulated, and they were significantly positively correlated with the number of oocytes retrieved, fertilized and high-quality embryos. It has been proved that defects in the synthesis of polyunsaturated fatty acids such as linoleic acid and linolenic acid lead to follicular arrest, oocyte atresia and infertility in female mice (42). Fatty acids are important energy source, mainly providing ATP for oocytes through β-Oxidation, and inhibition of β-Oxidation lead to meiosis arrest of oocytes and development failure after fertilization (43, 44). The decrease in fatty acids leads to DNA damage in granulosa cells and then accelerates cell apoptosis, which further reduces the quality of oocytes and embryos (45, 46). We know that the imbalance of oxidation/antioxidation is an important reason for DOR (47). There is evidence that arachidonic acid metabolism disorder is closely related to the reduction of ovarian reserve (18),and fatty acids and their derivatives participate in the regulation of cellular oxidative stress as antioxidants (48), which may be one of their roles in DOR.

In the FF of DOR patients, we also detected abnormal nucleotide metabolism, including decreased ribothymidine, N6-methyladenosine (m6A) and uric acid (UA). UA is the final product of human purine metabolism, and its physiological level can act as an antioxidant to reduce the damage of oxygen radicals and nitrite to cells (49). PCOS is closely involved in inflammation and oxidative stress, and some patients have obviously increased serum uric acid, which is related to the severity of the disease (50). Although no research has shown the role of UA in DOR, it can also be reasonably speculated that low UA levels in FF may cause increased oxidative stress and mitochondrial dysfunction and thus affect the growth and maturation of oocytes. N6-methyladenosine is one of the most common and abundant RNA modifications; it affects the stability, splicing and/or translation of modified RNA and therefore plays an important role in posttranscriptional regulation (51). A recent study found that in granulosa cells of aging ovaries, there was more m6A involved in the modification of mRNA, which damaged the normal process of mRNA degradation and accelerated the aging of granulosa cells (52). Mu. et al. also confirmed that m6A is indispensable in all stages of mouse follicular development, and oocytes lacking m6A cannot complete meiosis and form oosperms (53). With an increasing number of studies on m6A in granulosa cells and oocytes, m6A may become a new target for studying the pathogenesis and treatment of DOR.

## 5 Innovation and limitations

In the metabonomic study on the follicular fluid of patients with DOR, we used nontargeted metabonomic technology for the first time, obtained a more informative metabolic profile and established a diagnostic model based on metabolites. Our results provide data support for the study of the pathogenesis of DOR and the search for new diagnostic markers. However, our study still has some limitations. First, the sample size of the experimental groups is less than 30, which is only one small sample study. After expanding the sample, more metabolites with apparent differences may be obtained. Second, after obtaining the differential metabolites, we did not conduct cell experiments or animal experiments for validation or further study. Third, in subsequent research, targeted metabonomic studies could be carried out according to the metabolites obtained, which may be helpful to improve the metabolic pathway of DOR. Finally, our research is limited to metabonomics, and combining our results with genomics, transcriptomics and proteomics will hopefully promote research on the mechanism of ovarian reserve reduction.

## Data availability statement

The datasets presented in this study can be found in online repositories. The names of the repository/repositories and accession number(s) can be found in the article/**Supplementary Material**.

## Ethics statement

This study is a prospective clinical experiment that has been approved by the Ethics Committee of Wuhan University People’s Hospital (WDRY2018-K009), and all subjects signed an informed consent form. The patients/participants provided their written informed consent to participate in this study.

## Author contributions

JL, YW and TY conceived the original ideas. JL and YW collected samples and clinical data. JL, ZZ, YW and PZ conducted the metabolomics analysis. ZZ performed the statistical analysis. JL interpreted the data. JL and ZZ cowrote the manuscript. TY, SMC and QW supervised and revised the manuscript. All authors contributed to the article and approved the submitted version.

## Supplementary material

Supplementary Figure 1 Number of metabolites detected in different modes. HP: HILIC positive ionization mode; HN: HILIC negative ionization mode; RP: RPLC positive ionization mode; RN:RPLC negative ionization mode.

Supplementary Figure 2 PCA analysis and evaluation of quality control samples in different modes. a-b: HILIC (+) mode; c-d: HILIC (-); e-f: RPLC (+); g-h: RPLC (-).

Supplementary Figure 3 Correlation between differential metabolites.

Supplementary Table 1 Down-regulated metabolites in FF samples from DOR groups

Supplementary Table 2 Up-regulated metabolites in FF samples from DOR groups

Supplementary Table 3 Diagnostic models based on top5, top10, top 20 and total differential metabolites
