# Supplementary figures and images for: Metabonomic analysis of follicular fluid in patients with diminished ovarian reserve
Source: Front Endocrinol (Lausanne). 2023 Feb 27;14:1132621. doi: 10.3389/fendo.2023.1132621 (PMC10009106; doi:10.3389/fendo.2023.1132621)

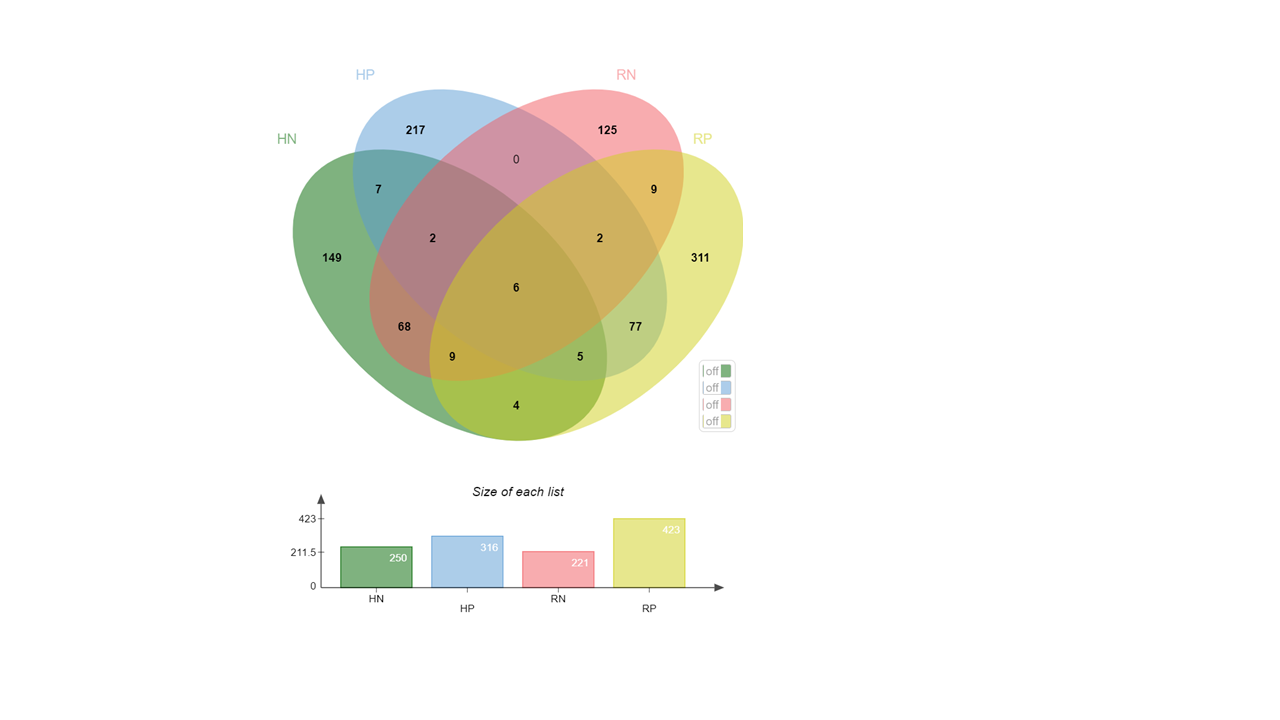

Supplement: Supplementary Figure 1 — Number of metabolites detected in different modes. HP: HILIC positive ionization mode; HN: HILIC negative ionization mode; RP: RPLC positive ionization mode; RN:RPLC negative ionization mode. [file Image_1.tif]

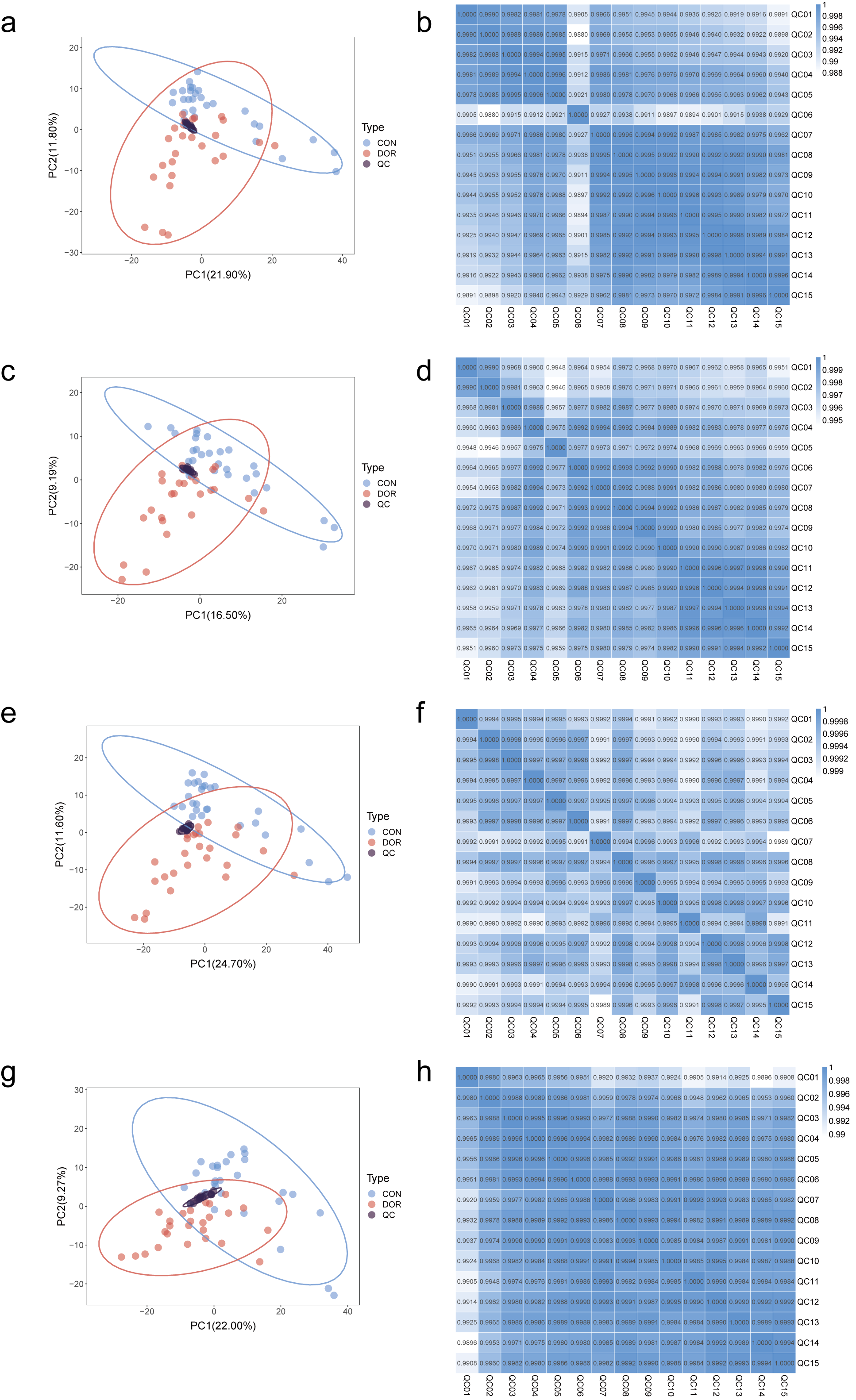

Supplement: Supplementary Figure 2 — PCA analysis and evaluation of quality control samples in different modes. a-b: HILIC (+) mode; c-d: HILIC (-); e-f: RPLC (+); g-h: RPLC (-). [file Image_2.tif]

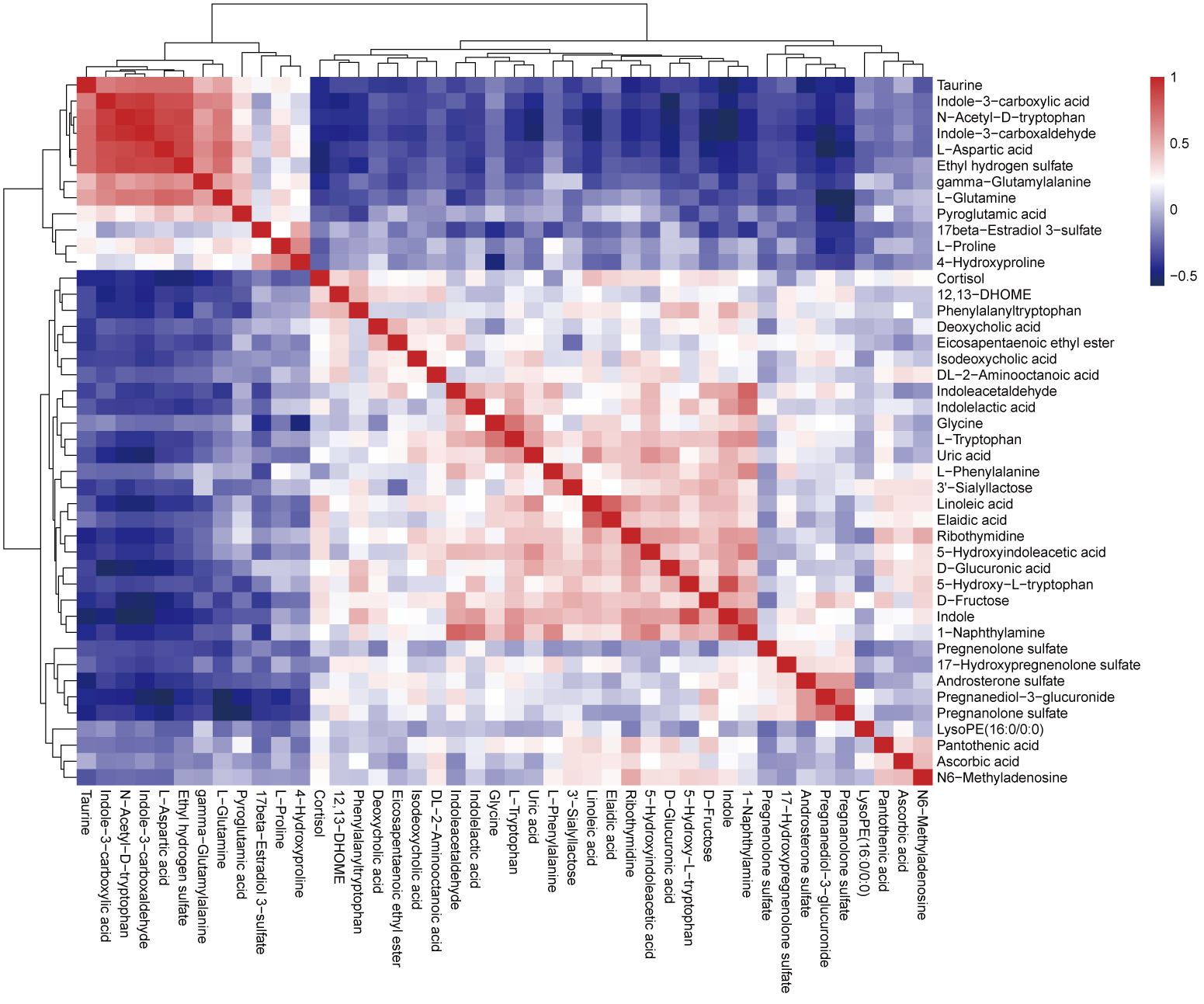

Supplement: Supplementary Figure 3 — Correlation between differential metabolites. [file Image_3.tif]
